# Development of a high throughput drug screening assay to identify compounds that protect oligodendrocyte viability and differentiation under inflammatory conditions

**DOI:** 10.1186/s13104-016-2219-8

**Published:** 2016-09-05

**Authors:** Karen D. Lariosa-Willingham, Elen S. Rosler, Jay S. Tung, Jason C. Dugas, Tassie L. Collins, Dmitri Leonoudakis

**Affiliations:** 1 Translational Medicine Center, Myelin Repair Foundation, Sunnyvale, CA 94085 USA; 2 Teva Pharmaceuticals, Biologics and CNS Discovery, Redwood City, CA 94063 USA; 3 Alios BioPharma, South San Francisco, CA 94080 USA; 4 Rigel Pharmaceuticals, South San Francisco, CA 94080 USA; 5 NGM Biopharmaceuticals, Inc., South San Francisco, CA 94080 USA

**Keywords:** Myelination, Oligodendrocyte, Cytokine, Interferon gamma, Tumor necrosis factor alpha, Protection, High throughput, Drug screening, Differentiation, Primary cell-based assay, Image analysis, Multiple sclerosis, Myelin basic protein

## Abstract

**Background:**

Newly proliferated oligodendrocyte precursor cells (OPCs) migrate and surround lesions of patients with multiple sclerosis (MS) and other demyelinating diseases, but fail to differentiate into oligodendrocytes (OLs) and remyelinate remaining viable axons. The abundance of secreted inflammatory factors within and surrounding these lesions likely plays a major inhibitory role, promoting cell death and preventing OL differentiation and axon remyelination. To identify clinical candidate compounds that may protect existing and differentiating OLs in patients, we have developed a high throughput screening (HTS) assay that utilizes purified rat OPCs.

**Results:**

Using a fluorescent indicator of cell viability coupled with image quantification, we developed an assay to allow the identification of compounds that promote OL viability and differentiation in the presence of the synergistic inflammatory cytokines, tumor necrosis factor α and interferon-γ. We have utilized this assay to screen the NIH clinical collection library and identify compounds that protect OLs and promote OL differentiation in the presence of these inflammatory cytokines.

**Conclusion:**

This primary OL-based cytokine protection assay is adaptable for HTS and may be easily modified for profiling of compounds in the presence of other potentially inhibitory molecules found in MS lesions. This assay should be of use to those interested in identifying drugs for the treatment of MS and other demyelinating diseases.

**Electronic supplementary material:**

The online version of this article (doi:10.1186/s13104-016-2219-8) contains supplementary material, which is available to authorized users.

## Background

Multiple sclerosis (MS) is a devastating neurological disease, affecting over 2 million patients worldwide, caused by autoimmune-mediated destruction of the myelin sheaths that insulate and protect axons in the central nervous system. Most MS patients initially present with the relapsing-remitting form of MS relapsing remitting multiple sclerosis (RRMS), in which cycles of immune-mediated axonal demyelination (relapse) are followed by periods of remyelination (remission). Over time, however, the majority of RRMS patients exhibit chronic, progressive decline in neurological function. This is believed to be the result of an accumulation of axonal damage, as well as an eventual loss of remyelination capacity [1]. Although current MS therapies for RRMS provide significant relief from relapse, none have yet been demonstrated to prevent disease progression and none have been effective in treating progressive forms of MS. All of the disease-modifying therapies approved for treatment of RRMS do so by targeting the pathologic immune response [2]. There are no therapies that function to directly protect or restore myelin, and identifying such therapies has been the focus of several groups in the field [3–6].

One approach undertaken by several groups seeking to identify new therapeutic agents, has been to screen small molecule libraries in an attempt to identify agents that accelerate differentiation of immature oligodendrocyte precursor cells (OPCs) into mature, myelin-producing oligodendrocytes (OLs) in the hope that this will accelerate remyelination of axons following immune attack. While it is logical to expect that increasing the number of mature oligodendrocytes will improve the rate and extent of axonal remyelination, it should be considered that the oligodendrocyte differentiation assays described above assess differentiation of OPCs into oligodendrocytes under healthy conditions. MS lesions contain a number of factors that are known to impair oligodendrocyte differentiation and reduce the viability of differentiating oligodendrocytes. We thus felt that it was important to develop assays useful for the identification of drugs that support oligodendrocyte differentiation under conditions mimicking disease. We therefore examined the effects of interferon-γ (IFNγ) in the presence tumor necrosis factor α (TNFα), a combination shown to be synergistically deleterious for other cell types in culture [7]. We observed that addition of TNFα alone had no ill effects on oligodendrocyte differentiation or viability. However, the presence of TNFα substantially increased the deleterious effects of IFNγ on both differentiation and viability of cultured oligodendrocytes, and did so at cytokine concentrations that were low enough to be relevant to the in vivo disease state. Using this information, we developed an assay suitable for rapid high throughput screening, to identify drugs that protect differentiating oligodendrocytes from cytokine toxicity. We used this assay to screen the NIH clinical collection (NCC) library (a library of 727 drugs that have been approved for the treatment of various indications), and report here the identification of several FDA-approved drugs that sustain viability and differentiation capacity of oligodendrocytes in the presence of IFNγ and TNFα in vitro. This assay will be useful for screening novel compound libraries and is adaptable to screening compounds in the presence of other factors present in MS lesions.

## Findings

### Development of an oligodendrocyte protection assay in the presence of inflammatory cytokines

We developed an assay to identify compounds that would protect differentiating OLs under adverse conditions, such as those present in MS lesions. Pro-inflammatory cytokines are known to have detrimental effects on OL differentiation and viability [8–11] and thus we incorporated the adverse challenge of two prominent components of the inflammatory cascade, TNFα and IFNγ, into our assay conditions. TNFα has been demonstrated to be present in MS lesions along with IFNγ and its expression levels change during different stages of the disease [12–14].

**Fig. 1:**
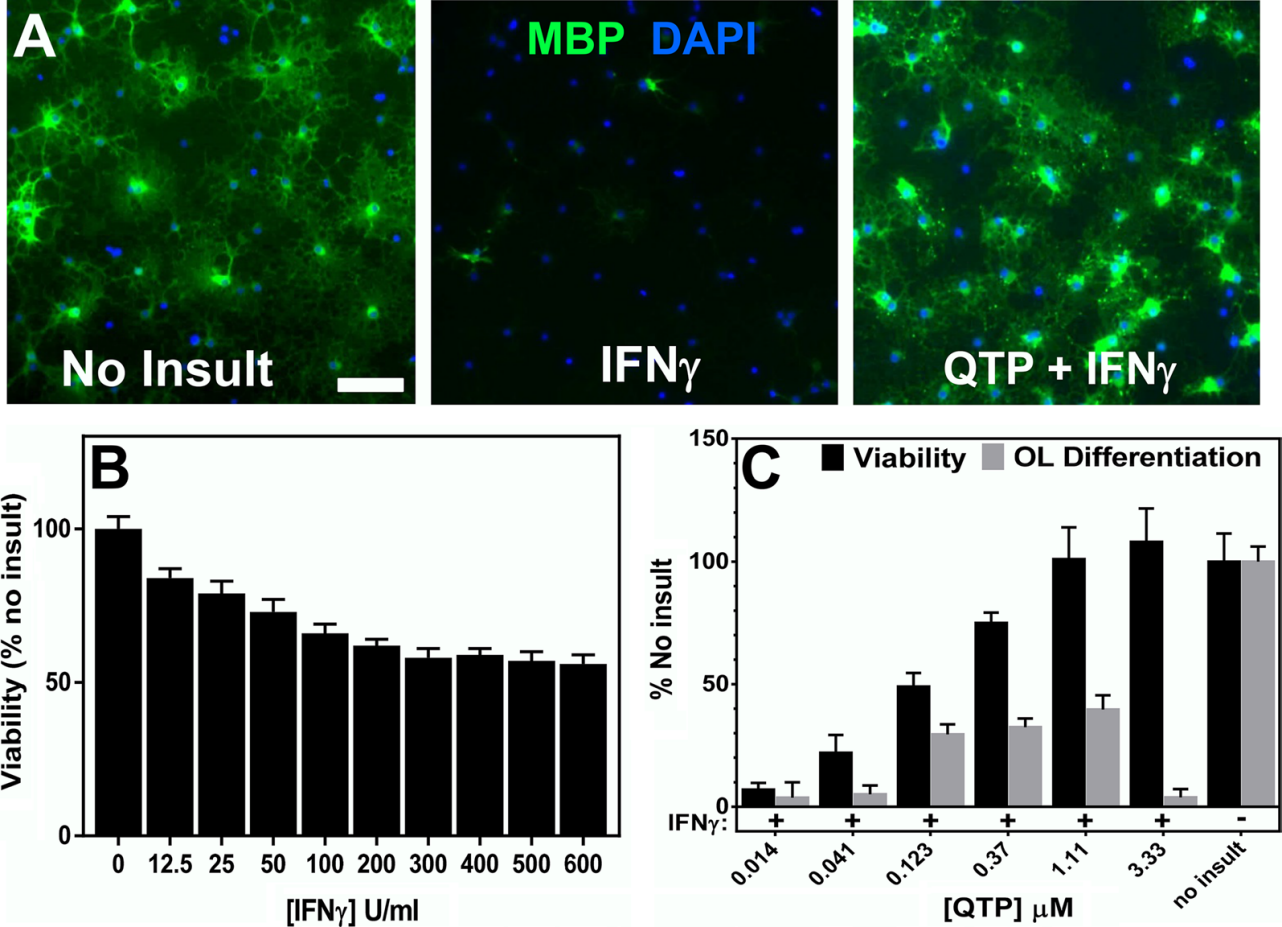
IFNγ reduces OL viability and inhibits differentiation. **A** Expanded OPCs were plated in 96 well plates in DMEM differentiation media for 24 h and either treated for 1 h with 1.1 µM quetiapine (QTP) or vehicle (0.1 % DMSO), followed by 48 h of 200 U/ml INFγ insult. No Insult was vehicle treated with no IFNγ insult. OLs were immunostained for MBP (*green*) and nuclei stained with DAPI (*blue*).* Bar* 200 μM. **B** 24 h after plating, differentiating OLs were treated with increasing concentrations of INFγ for 48 h. alamarBlue® (AB) fluorescence was quantified to determine cell viability. **C** 24 h after plating, differentiating OLs were treated with increasing concentrations of QTP for 1 h followed by 48 h treatment with 200 U/ml IFNγ. Cell viability was measured by quantification of alamarBlue® fluorescence. Image quantification of anti-MBP immunostaining of the same cultures was used to measure OL differentiation

**Fig. 2:**
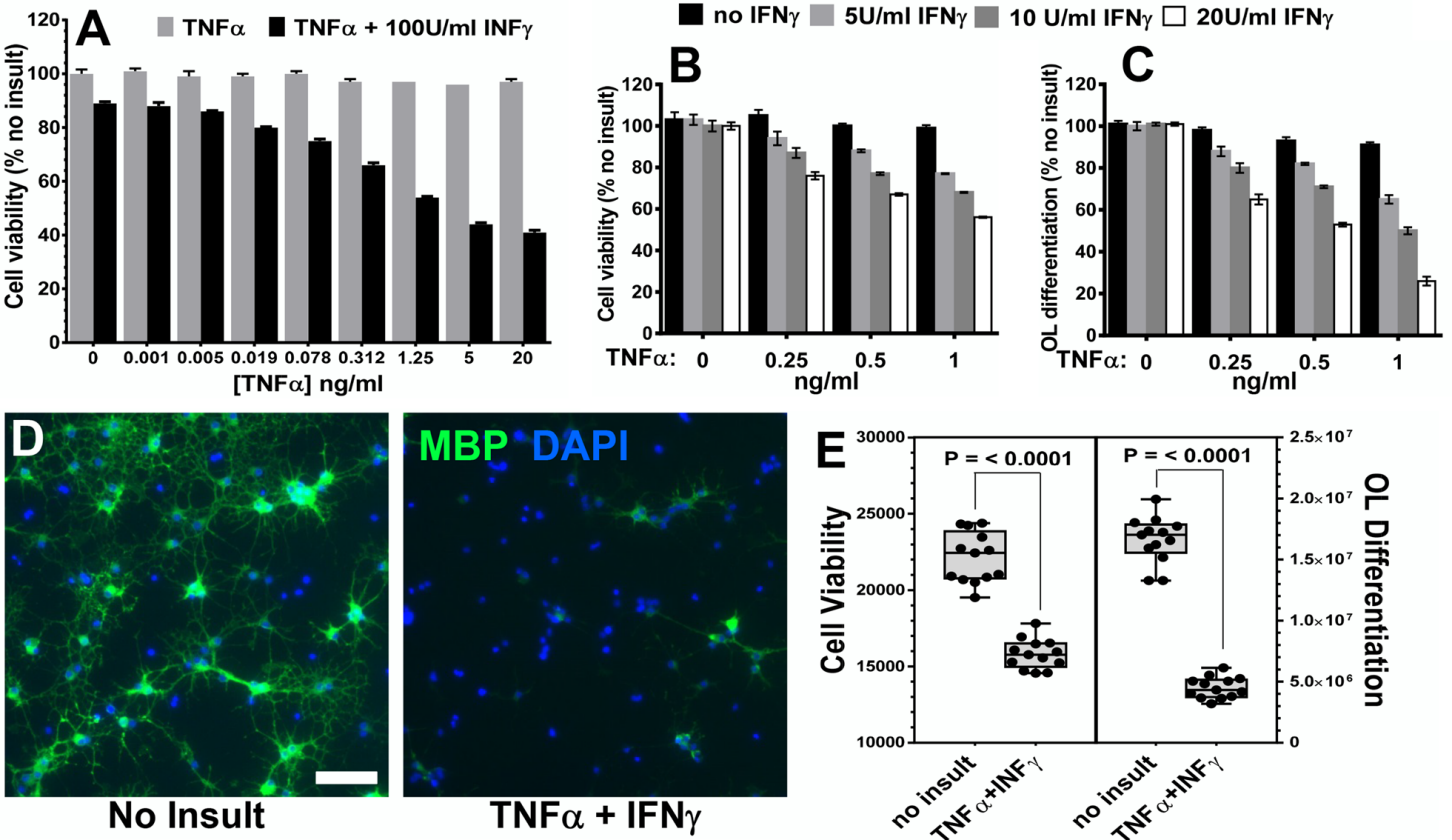
TNFα synergizes with IFNγ causing reduced OL survival and inhibiting differentiation. Expanded OPCs were plated in DMEM differentiation media for 24 h and then treated with various combinations cytokines for 48 h. **A** Differentiating OLs were treated with increasing concentrations of TNFα alone (*gray bars*) or increasing concentrations of TNFα + 100 U/ml INFγ (*black bars*) and alamarBlue® (AB) fluorescence was quantified to determine cell viability. **B** Cells were treated as in A with increasing concentrations of TNFα alone (*black bars*) or TNFα + 5, 10, or 20 U/ml INFγ (*light gray*, *dark gray*, or *white bars* respectively) and AB fluorescence was quantified to determine cell viability. **C** Image quantification of anti-MBP immunostaining of the same cultures as in B was used to measure OL differentiation. **D** Comparison of MBP expression in untreated (No insult) or 10 U/ml INFγ + 1 ng/ml TNFα treated differentiating OLs. **E** Viability and OL differentiation raw values of no insult versus 10 U/ml INFγ + 1 ng/ml TNFα demonstrate the reproducibility of the combined cytokine toxicity. Coefficient of variation (CV) values for cell viability were 7.47 and 6.14 % and for OL differentiation were 11.37 and 19.16 % for No insult or TNFα+INFγ, respectively. CV values <20 % were considered in the acceptable range

In vitro expanded OPCs were plated in PDL-coated 96-well plates at 10,000 cells/well in differentiation media supplemented with T3, but without PDGF in order to promote OL differentiation. These minimally expanded OPCs differentiated into OLs within 3 days in the presence of T3 as determined by imaging of MBP expression (Figs. 1A, 2D).

To determine the effects of inflammatory cytokines on actively differentiating OLs, we initially tested the effects of exposure to IFNγ alone on the viability of T3-induced differentiating OLs, an insult previously shown to cause apoptosis of OLs [8]. Following 48 h of IFNγ exposure, the viability of cells was determined by alamarBlue^®^ (AB—a fluorescent indicator of metabolic activity) fluorescence quantification. At least 200 Units/ml of IFNγ was required to generate a 20 % reduction in differentiating OL viability when added alone (Fig. 1B). We used this concentration to test whether we could identify small molecules that could rescue OLs from IFNγ toxicity. We tested the confirmed hit compounds identified in an acute OL differentiation assay (see [15]). We added test compounds 24 h following OPC plating and 1 h prior to the addition of INFγ insult to give compounds the best chance for efficacy; cells were assayed 48 h later for viability with AB. Since AB fluorescence is produced by incubation with live cells and is non-toxic, we were able to wash, fix, and immunostain the same cultures for MBP expression (see “Methods” section) and quantify MBP expression. Figure 1A shows the decrease in MBP immunostaining intensity caused by 200 U/ml IFNγ. Additional file 1: Table S1 includes the EC_50_ values for protection from IFNγ-induced toxicity and restoration of OL differentiation (image quantification of MBP expression—see [15]) calculated for each compound tested. Figure 1C shows the dose response relationship of OL protection from toxicity induced by 200 Units/ml IFNγ of one of the most efficacious compounds tested, quetiapine (QTP). 1.1 µM QTP was determined to be the most efficacious concentration in protecting OLs from IFNγ toxicity (Fig. 1B) as well as for preserving MBP expression (Fig. 1C).

In an attempt to increase the cytokine protection window, we tested TNFα alone, up to 20 ng/ml, which had no measurable impact on viability of OLs (Fig. 2A). Since it is known that IFNγ and TNFα can combine to have synergistic effects and are both present in MS lesions [8, 16], we titrated each cytokine against the other to determine possible synergistic effects for use in the OL protection assay. When TNFα was titrated against a fixed concentration of 100 U/ml IFNγ, the combination of 100 U/ml IFNγ + 1.25 ng/ml TNFα resulted in a drop in OL viability of 46 % vs. only 11 % for 100 Units/ml of IFNγ alone (Fig. 2A). To further optimize the synergistic combination of TNFα and IFNγ we narrowed the range of concentrations and measured both OL viability and differentiation. We were able to titrate IFNγ down to 10 U/ml in combination with 1 ng/ml TNFα and still reproducibly obtain ~30 % reduction of OL viability (Fig. 2B, E). This cytokine combination also resulted in ~50 % reduction in OL differentiation as determined by quantification of MBP expression (Fig. 2C–E). Concentrations of TNFα above 1 ng/ml resulted in toxicity that reduced MBP to undetectable levels.

We used this newly developed cytokine protection assay (TNFα + IFNγ) to evaluate the activity of the hit compounds identified in the acute OL differentiation screen (see [15]). We generated dose response curves and EC_50_ values for each compound for viability and OL differentiation (Additional file 2: Figure S1; Table 1). Additionally, Additional file 2: Figure S1 shows the chemical structures of confirmed hit compounds and representative MBP/DAPI images taken from the screen. All compounds that rescued OL differentiation also increased cell viability. However, three compounds were negative for viability promoting activity, showing very little cytokine protection activity or OL differentiation (<20 % rescue; citalopram, esmolol, and dofetilide). One class of compounds having anti-fungal activity (bifonazole, clotrimazole, econazole) sustained viability of OLs but did not preserve OL differentiation in the presence of cytokines (Additional file 2: Figure S1F^1^, S1G^1^, S1H^1^). This indicates that the mechanism of cytokine protection can be separated from the promotion of differentiation in the presence of inflammatory cytokines.

**Table 1:** Confirmed cytokine protection hits from the NCC library screen

Drug class	Compound	AB screening (% quetiapine)	AB EC_50_ (μM)	MBP EC_50_ (μM)	Hit in OL diff^a^
SERM	Raloxifene	92	0.03	0.05	**•**
	Toremifene	73	0.02	0.01	**•**
	Tamoxifen	117	0.02	0.01	**•**
	Mestranol	93	0.92	0.79	
	Clomiphene	52	0.01	0.01	
Tricyclic anti-depressants	Perphenazine	96	0.01	0.03	**•**
	Fluphenazine	91	0.02	0.02	**•**
	Prochlorperazine	110	0.04	0.04	**•**
	Trifluoperazine	155	0.02	0.03	**•**
	Quetiapine	130	0.15	0.13	**•**
Non-tricyclic anti-depressants	Perospirone	85	0.22	0.15	**•**
	Bupropion	71	3.89	3.86	**•**
	Bifemelane	78	0.20	0.26	
	Cinanserin	64	0.97	1.59	
	Trazadone	55	7.18	5.42	
Muscarinic	Clemastine^c^		0.14	0.14	**•**
	Benztropine	123	0.17	0.14	**•**
	Donepezil	95	0.46	0.74	**•**
	Vesamicol	151	1.83	2.26	**•**
	Oxybutynin	92	0.67	0.54	**•**
	Propantheline	91	2.95	1.25	
Adrenergic	Salmeterol	55	0.22	0.20	**•**
	Betaxolol	52	5.56	6.21	**•**
Ion channel	Ifenprodil	162	0.36	0.52	**•**
	Benproperine	99	0.43	0.12	**•**
	Proxymetacaine	98	1.23	0.78	**•**
	DMPP	54	2.18	2.84	**•**
Anti-fungal	Bifonazole	105	0.44	^b^	**•**
	Clotrimazole	171	0.77	^b^	
	Econazole	90	0.34	^b^	
Antidiabetic	Pioglitazone	56	7.34	^b^	
LO inhibitor	MK886	51	0.31	0.19	
Histamine H1 Antagonists	Hydroxyzine Pamoate	106	0.15	0.11	
	Meclizine	97	0.12	0.10	
Retinoids	All-trans-retinoic acid	128	0.88	0.44	
	Acitretin	69	2.23	1.09	

*LO* lipooxygenase

^a^Hit in acute OL differentiation assay (as determined in [15])

^b^Inconsistent, weak OL differentiation activity

^c^Not in NCC library

**Fig. 3:**
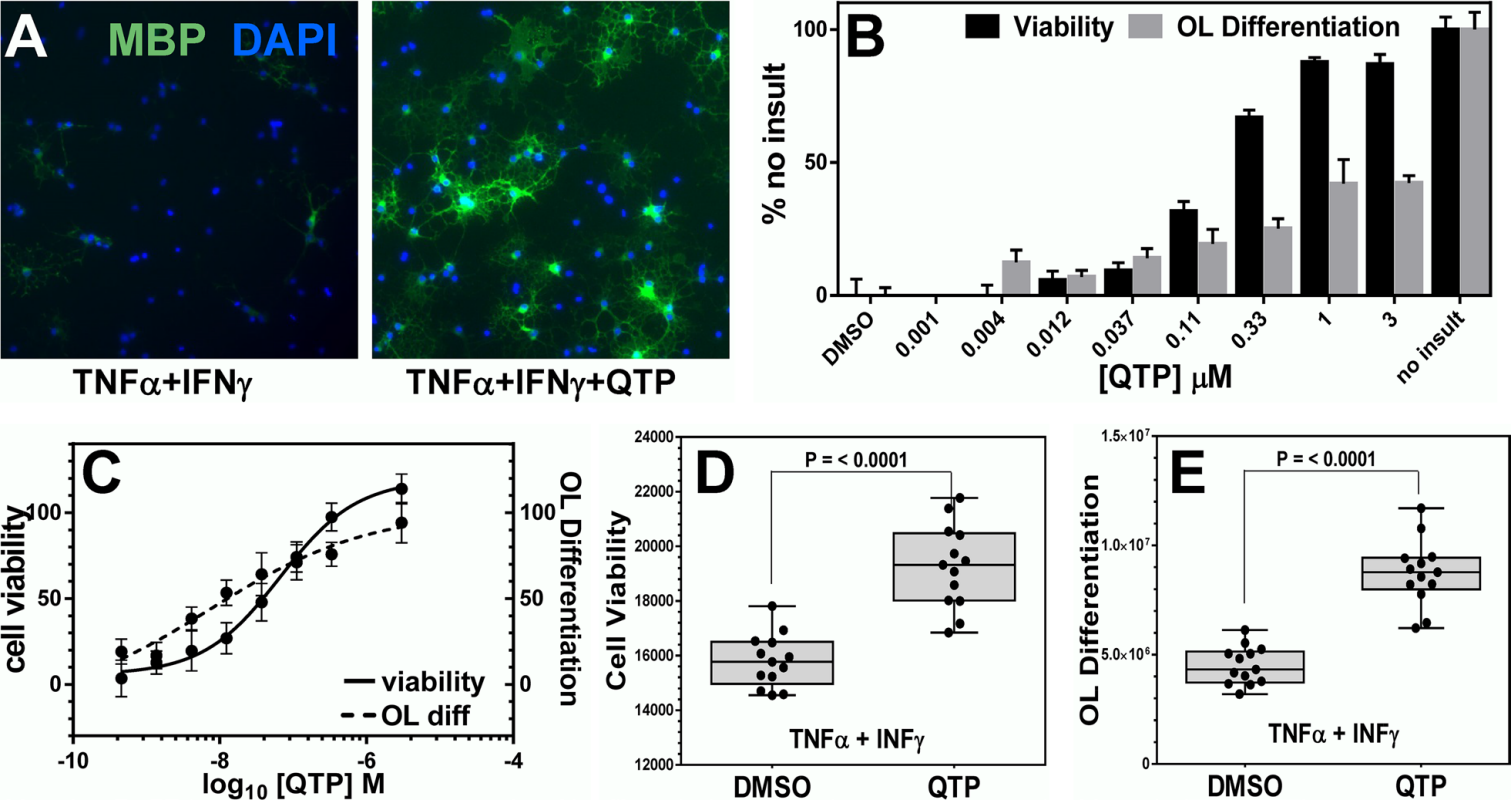
Quetiapine (QTP) reproducibly rescues viability and OL differentiation in the presence of cytokine-mediated toxicity. Expanded OPCs were plated in 96 well plates in DMEM differentiation media for 24 h and either treated for 1 h with 1.1 µM quetiapine or 0.1 % DMSO vehicle, followed by 48 h of 1 ng/ml TNFα + 10 U/ml INFγ insult. **A** Comparison of MBP expression in TNFα+INFγ or TNFα+INFγ+QTP treated differentiating OLs. OLs were immunostained for MBP (*green*) and nuclei stained with DAPI (*blue*). **B**, **C** The dose–response relationships and curves were determined and scaled to DMSO (0 % and no insult (100 %). The EC_50_ values for QTP were calculated to be 157 nM (n = 4, mean ± SEM) for cellular viability and 125 nM (n = 4, mean ± SEM) for OL differentiation. **D**, **E** Viability and OL differentiation raw values of negative control DMSO and positive control QTP demonstrate the reproducibility of QTP protection from cytokine toxicity (n = 13, mean ± SEM). Coefficient of variation (CV) values for cell viability were 6.14 and 7.92 % and for OL differentiation were 19.16 and 17.21 % for DMSO and QTP, respectively. CV values <20 % were considered in the acceptable range

We then assessed the ability of QTP to protect OLs from the combined 1 ng/ml TNFα and 10U/ml IFNγ toxicity. QTP preserved OL differentiation in the presence of both 1 ng/ml TNFα and 10 U/ml IFNγ demonstrated by imaging of MBP immunostaining (Fig. 3A). Figure 3B, C shows the dose–response relationships for QTP in both parameters of the cytokine protection assay. The EC_50_ values were determined to be 157 nM for viability protection and 125 nM for OL differentiation protection. From this data, we chose 1.1 µM as a positive control assay concentration to be used in subsequent assay screens. 1.1 µM QTP reproducibly rescued differentiating OLs from cytokine-induced toxicity by an average of 90 % (Fig. 3D) and restored their ability to differentiate by 60 % (Fig. 3E).

### NCC Library screening for compounds that protect differentiating OLs from combined INFγ and TNFα insult

Prior to library screening, NCC library compounds were prescreened at three concentrations for OL toxicity (see [15]). This allowed for the reduction of toxic concentrations and to potentially detect active compounds active at lower doses.

**Fig. 4:**
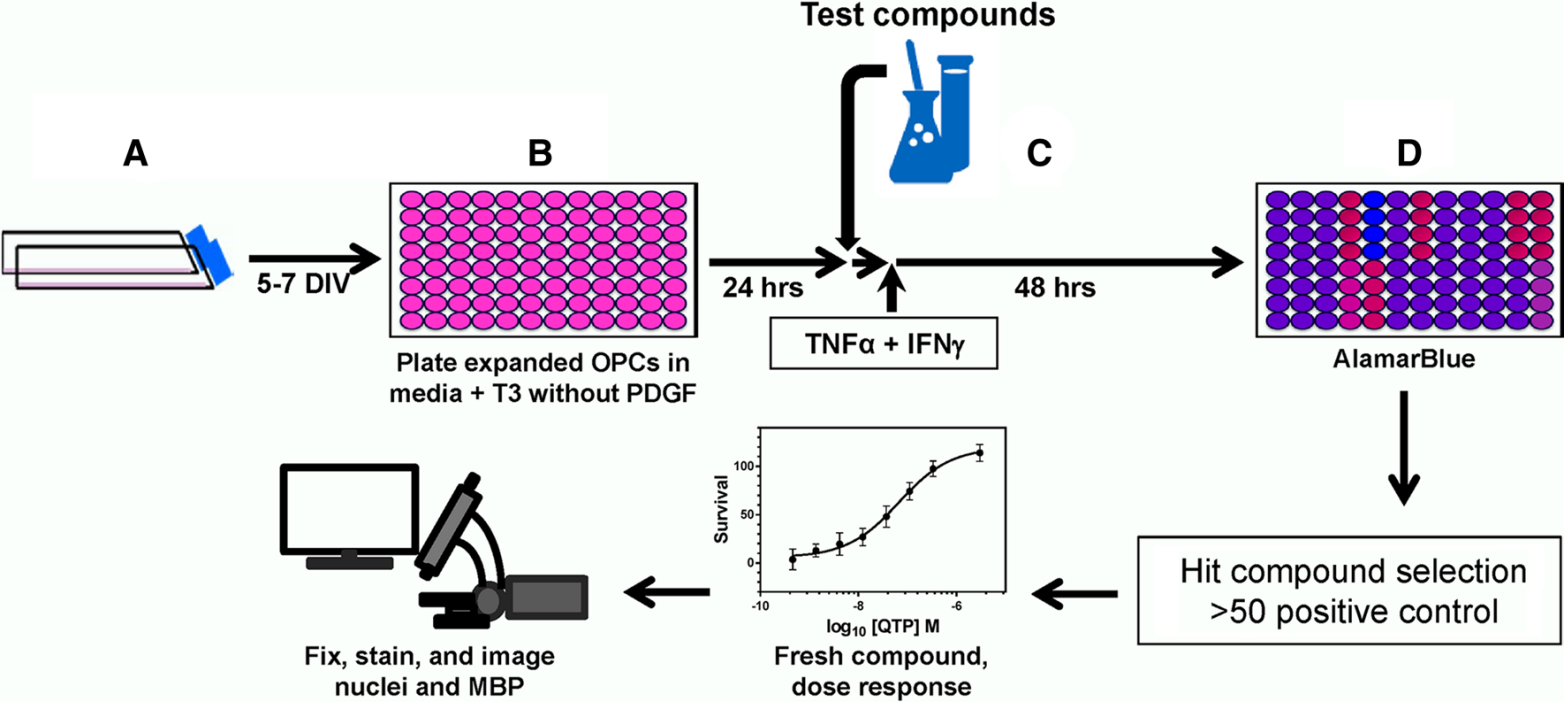
Flow scheme of the cytokine OL protection assay. A primary O4-positive OPCs were isolated for P6–P7 neonatal rat brains. *A* OPCs were expanded in flasks for 5–7 days in defined media supplemented with PDGF. *B* Expanded OPCs were plated in 96-well plates in defined media supplemented with T3, but without PDGF. *C* Test compounds were added following a 24-h incubation period followed 1 h later by addition of cytokine challenge (1 ng/ml TNFα+10 U/ml IFNγ). *D* After 48-h culture, alamarBlue® (AB) is added and AB fluorescence used to determine viability and metabolic activity of the cells. After selection of hit compounds (>50 % of the positive control, 1.1 µM QTP), fresh compound from a new source was obtained and the dose response relationship and EC_50_ values of each compound were determined. Following AB read out of the dose response, cells were fixed, immunostained, and images quantified to determine OL differentiation (MBP expression)

To reduce time, reagent and labor costs, we decided to screen the NCC library using the viability detection portion of the cytokine protection assay, as we observed that OL differentiation was only rescued if cell viability was rescued in profiling the hits from the acute OL differentiation assay. Figure 4 shows the flow scheme for the cytokine protection assay. OPCs were purified, expanded in the presence of PDGF and plated in 96 well plates as described above. Differentiation was initiated by PDGF withdrawal and T3 supplementation. Following a 24-h incubation period, test compounds were added (at two different concentrations initially determined from the OL toxicity assay) followed 1 h later by addition of 1 ng/ml TNFα + 10 U/ml IFNγ. After 48-h incubation in the presence of cytokines, AB was added to cells and the fluorescence quantified to determine cell viability. Each compound concentration was screened in triplicate with data scaled relative to 0.1 % DMSO as the negative control and 1.1 µM QTP as the positive control. A no insult control was also included to assess the consistency of cytokine toxicity.

**Fig. 5:**
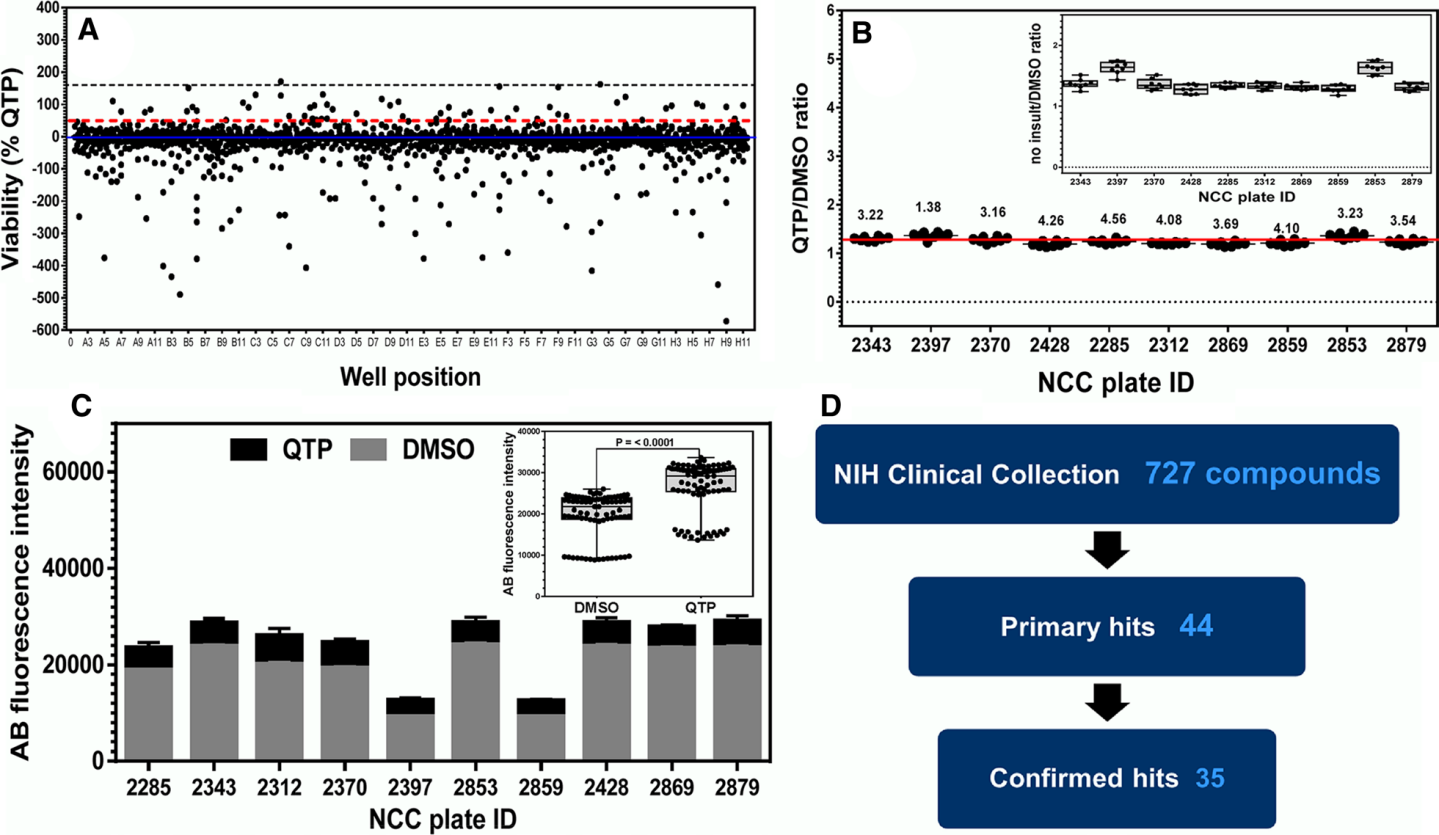
Analysis of the cytokine OL protection assay of the NCC compound library. **A** Dot plot representation of the entire high-throughput screening data set used to identify promoters OL survival. The mean response is indicated by the* blue solid line*. The *black dotted line* delineates the value of three standard deviations above the mean. The *red dotted line* in delineates the ≥50 % positive control selection criteria. **B** Scatter plot representation of the high-throughput screening assay window. Using the QTP/DMSO ratio as the window of OL survival, the ratio of the positive (QTP) to negative (DMSO) controls is depicted in the scatterplot. The red line delineates the mean average ratio value for the entire NCC library screen = 1.3. The numbers above the scatter indicate the coefficient of variation (CV) for each plate. CV values <20 ± 5 were considered in the acceptable range. *Each point* is an average value from each screening plate (n = 4 wells, mean ± SEM).* Inset* depicts consistency of cytokine insult for the entire library with a mean average value of 1.4. **C** An overlapping representation of raw AB values of DMSO and QTP for each library screening plate. The *inset* depicts a statistical analysis of the raw AB values for the entire NCC library. **D** Summary tally of hits from the NCC library screen

The criterion for a hit compound was protection of differentiating OLs from cytokine toxicity that was at least 50 % of the positive control, 1.1 µM QTP level or above. Similar to the OL differentiation assay, we included in our analysis the visualization of three SDs above the mean (Fig. 5A). Alongside this, we show the 50 % QTP threshold line (Fig. 5A). Control QTP versus DMSO values from the entire screen for OL differentiation were highly statistically significant per assay plate (Fig. 5B, C) as well as when averaged over entire screen (Fig. 5C, inset) indicating an acceptable screen window and reproducibility. In a screen of 727 FDA-approved drugs, we identified 44 actives (6 % primary hit rate) able to protect differentiating OLs from cytokine toxicity at one or both concentrations tested (Fig. 5D). Additional file 3: Table S2 includes the data for all compounds tested in the cytokine protection screen.

### Independent confirmation and validation of identified hits

In order to confirm the activity of the primary hits identified in the cytokine protection assay, we purchased 40 fresh compounds from independent vendors and performed a dose response analyses (4 compounds were excluded from follow up). We confirmed 36 active compounds (90 % hit confirmation rate) and determined the EC_50_ values for both viability (AB fluorescence quantification) and OL differentiation (quantification of MBP immunostaining) protection in the presence of inflammatory cytokines (Table 1).

## Discussion

In an effort to identify possible therapeutic candidate compounds for multiple sclerosis and other demyelinating diseases, we have established a high throughput assay that can be used to rapidly screen large compound libraries for molecules that protect differentiating OLs from the deleterious effects of inflammatory cytokines. We reasoned that the differentiation and viability of OLs at the site of a demyelinating lesion may be compromised as a result of exposure to a variety of inhibitory and/or toxic stimuli, including inflammatory cytokines. We used this assay to screen the NCC library, assessing each compound’s ability to protect differentiating OLs from the combinatorial effect of INFγ and TNFα toxicity. We identified and confirmed 35 hit compounds establishing their potency and efficacy. To the best of our knowledge, this is the first high-throughput screen designed for the purpose of assessing OL differentiation and survival of differentiating OLs under inflammatory conditions, such as those that would prevail in an MS lesion.

There are a number of advantages of adapting this assay for the purpose of screening compound libraries for compounds that protect OLs from toxic insult. The robustness, scale and reproducibility are amenable to HTS drug discovery. The assay uses minimally-passaged primary rat cells, obviating the need to produce large numbers of OPCs either through multiple passages or by derivation from large-scale stem cell cultures. We designed the assay to preserve primary cell, in vivo-like characteristics in culture by limiting subculture to a single expansion. Thus, we avoided undesirable changes in cellular phenotype (e.g. gene expression and/or unstable karyotypes) which multiple cell passages may introduce [3, 17–19]. We have also demonstrated that additional endpoints combined within the same assay can introduce interesting predictive cellular phenotypes. Although the OL protection assay could be scored using OL differentiation as an image-based endpoint for identification of drugs that support OL differentiation in the presence of inflammatory mediators, we have demonstrated that OL viability is an endpoint that is highly predictive of differentiation (33 of 36 hit compounds). Viability can be rapidly and inexpensively assessed using alamarBlue®, permitting much higher throughput than the image-based OL differentiation assays. Moreover, alamarBlue® reagent is easily removed, permitting subsequent staining and imaging for OL differentiation if desired.

It is important to note that we have only evaluated OL differentiation in the presence of just two cytokines, but this delineates just one of many relevant, potentially detrimental, cytokine combinations. This OL protection assay is highly flexible and should be useful for evaluating a wide variety of other factors, such as examining the effects of other cytokines known to be present in MS lesions (alone and in combination) and other inflammatory mediators such as myelin debris/breakdown products that may have detrimental effects on OLs.

### Confirmed hits group into multiple classes

In a companion screen of the NCC library using an OL differentiation assay (see [15]), we identified 27 hit compounds capable of promoting the differentiation of OPCs into OLs. Twenty-two of these compounds also protected OLs from inflammatory cytokine insults (Table 1). Intriguingly, an additional 16 compounds were identified that specifically rescued OL differentiation and promoted survival from cytokine toxicity. We grouped these hit compounds into the following categories: (1) muscarinic acetylcholine signaling, selective estrogen receptor modulators (SERMs), tricyclic antidepressants, non-tricyclic antidepressants, adrenergic receptor signaling, which were hits in both the OL protection assay and the OL differentiation assay and (2) antidiabetic, anti-retroviral, lipoxygenase inhibitor, histamine H1 antagonists and the retinoids which were only identified in the OL protection screen (Table 1). Within these classes emerged hit compounds that have relevance to MS and may be repurposed for therapeutic use in the treatment of MS.

### Relevance to multiple sclerosis

We chose to screen the NCC library because it is a small, focused FDA-approved set of compounds with great clinical potential. Additionally we chose to screen at multiple concentrations in hopes of identifying compounds that are more efficacious at lower concentrations. While this added time to the process, we felt that this increase in screening time was more than offset by rapid progress to clinical trials for any such identified drugs that are FDA-approved.

There are many examples where repurposed drugs are making an impact in the field of MS. A few of note are BG-12 (Tecfidera®), a modification of a psoriasis drug; fingolimod (Gilenya®), originally developed for transplantation and alemtuzamab (Lemtrada™), originally an oncology drug. We found that a fair number of our hits aligned with current MS repositioning efforts in terms of patents, preclinical work, and clinical trials and are briefly cited here.

QTP, our positive control for OL protection, has been reported in the literature to provide protection from the development of EAE symptoms and cuprizone insult, both rodent models of demyelinating disease, as well as preventing myelin loss and promoting OL differentiation in vivo [20–24].

The SERMs represent a promising new class of compounds that protect OLs from cytokine toxicity and promote OL differentiation. Modulation of the estrogen receptor has been demonstrated to be effective in preventing demyelination in vivo and to stimulate differentiation and remyelination [25–28]. Specifically, raloxifene (Evista®) was shown to be effective in suppressing EAE [29]. Additionally, the pregnancy hormone estriol (Trimesta™), has shown efficacy in a clinical trial of MS reducing the lesion numbers and volumes [30]. This indicates that modulation of the estrogen receptor pathway may be a viable target for OL differentiation and protection. Interestingly, sexual dimorphism has been noted within OL preparations with OLs derived from males and females separately responding differently to sex hormones [31]. Our OL preparations were derived from randomly selected P7 rat pups and therefore the response to some sex hormone signaling pathways may show differences from OLs isolated from one sex or the other.

A number of hits that we identified are covered in patents for oligodendrocyte directed differentiation. Notably trifluoperazine (Stelatzine®), benztropine (Cogentin®), ipatropium (Atrovent®), and clemastine (Tavist®), (US 20140038949 A1) are patented for the use of neurotransmitter receptor modulating agents for inducing OL differentiation, as well as methods for treating subjects with such agents in a demyelinating disease.

Other hits have been evaluated or will soon be tested in human MS trials. QTP (Seroquel XR®), an antipsychotic approved for schizophrenia is currently in clinical trials to determine tolerability to people with RRMS and progressive MS (Clinicaltrials.gov identifier: NCT02087631). Selective serotonin re-uptake inhibitors (SSRIs) such as nitalapram (Celexa®) and escitalopram (Lexapro®) have also been in human MS trials. Nitalapram is one of three other SSRIs tested as an add-on with fingolimod, a current immunomodulatory treatment for MS (REGAIN Trial NCT01436643) in RRMS patients with depression. Escitalopram, has been in a study to test improvement of depression in patients of MS or ALS (NCT00965497). Donepezil (Aricept®), used as a treatment for dementia, has been used in a study to improve cognitive dysfunction in MS patients (NCT00315367) [32–34]. Oxybutynin (Ditropan XL®**)** is used currently in symptom management for MS to treat overactive bladder in MS (SONIC trial NCT00629642). Clemastine fumarate (Tavist®) originally approved as a first-generation antihistamine, is active in a current study to assess the drug as a remyelinating agent is RRMS patients (ReBUILD trial NCT02040298).

In preclinical studies, some of the compounds we identified as having protective properties show great promise in MS. The peroxisome proliferator-activated receptor gamma agonist, pioglitazone (Actos®—a drug used for diabetes), shows protective effects in the brain, delays onset and severity in rodent EAE models [35, 36] and is used as an add-on therapy for RRMS for treatment of nerve pain [37]. MK886, a lipoxygenase inhibitor, may be a useful therapeutic disease modifying treatment in MS, and it has been demonstrated to attenuate neuron inflammation, motor dysfunction and axonal damage in the mouse cuprizone-induced demyelination model [38]. Retinoic acid (Vesanoid®), an anti-cancer chemotherapy drug, has been cited to have a protective role in vitro against neuro-inflammatory insults in vitro [39].

The antihistamines, hydroxyzine (Atarax®) and meclizine (Antivert®), identified from the OL protection screen are currently used for symptom management in MS. Hydroxyzine is used for alleviation of sensory symptoms in MS and cited in a published pilot clinical trial in MS patients [40]. Meclizine, used to treat vertigo, motion sickness and nausea, also treats those symptoms MS, as well as vomiting and dizziness symptoms.

To our knowledge, the remainder of our confirmed hits has not been tested in any demyelinating diseases. Future studies should focus on testing these compounds both in vitro with human cells as well as in vivo models of MS.

## Conclusion

Here, we have described a large-scale primary cell-based drug discovery OL protection assay, with paradigms relevant to the MS environment and performed with industry rigor. We hope that other groups will find this assay helpful for comprehensively examining the effects of these factors on oligodendrocyte differentiation and health, and for developing drug screens that more closely mimic the pathology of the MS lesion.

## Methods

High glucose Dulbecco’s Modified Eagle Medium (DMEM), Neurobasal medium (NB), Earle’s Balanced Salt Solution (EBSS), l-glutamine, Fetal Bovine Serum (FBS), penicillin/streptomycin, sodium pyruvate, diamidino-2-phenylindole, dilactate (DAPI) were purchased from Life Technologies (Carlsbad, CA, USA). Normal goat serum, forskolin, triiodothyronine (T3, thyroid hormone), vitamin B12, hydrocortisone, d-biotin, apotransferrin, putrescine, progesterone, sodium selenite, poly-d-lysine (PDL), recombinant human (rh) insulin, bovine serum albumin, *N*-acetyl-l-cysteine, sodium phosphate (mono- and dibasic), sodium chloride, tris base, l-lysine, sodium azide, tunicamycin and DMSO were obtained from Sigma-Aldrich (St. Louis, MO, USA). Trace elements B, Dulbecco’s Phosphate Buffer Saline (dPBS) and trypsin 0.05 %-EDTA were purchased from Mediatech, Inc. (Manassas, VA, USA). Human ceruloplasmin was purchased from EMD Millipore (Billerica, MA, USA). Recombinant rat (rr)CNTF, rrIFNγ, rrTNFα, rhNT-3 and rhPDGF-AA were purchased from PeproTech (Rock Hill, NJ, USA). DNase, papain and ovomucoid inhibitor were purchased from Worthington Biochemical Corporation (Lakewood, NJ, USA). Paraformaldehyde solution was purchased from Electron Microscopy Science (Hatfield, PA). alamarBlue® (AB) was purchase from AbD Serotech (Killington, UK). Falcon TC 96-well plates were purchase from Corning (Corning, NY). Additional file 4: Table S3 lists the primary antibodies and their dilutions used in this study.

### Compounds

All compounds in the NIH Clinical Collection (NCC) library were supplied in DMSO at 10 mM in 96-well plates. Hit compounds were purchased as powders and stock solutions were dissolved in DMSO to 10 mM (see Additional file 3: Table S2 for complete listing of compounds).

### Isolation and expansion of neonatal rat OPCs

All animal work was carried out in strict accordance with the recommendations in the Guide for the Care and Use of Laboratory Animals of the National Institutes of Health. The protocol was approved by the Institutional Use and Care and Use Committee at the Molecular Medicine Research Institute (Sunnyvale, CA). Animals used for OPC isolation in these studies were euthanized by decapitation.

OPCs from brains of P6–P7 Sprague–Dawley rat pups (Charles River, Wilmington, MA, USA) were enriched by immunopanning as previously described [41]. Briefly, single cell suspensions were obtained from papain-digested neonatal brains and depleted of mature glial cells by successive panning with RAN-2- and GalC-coated plates, followed by enrichment for OPCs using O4-coated plates.

In vitro expanded OPCs were used in toxicity and cytokine protection assays. Acutely enriched OPCs were seeded into PDL-coated tissue culture flasks at 1000–2000 cells/cm^2^ in differentiation media (DMEM, 100 U/ml Penicillin/100 µg/ml Streptomycin, 2 mM l-Glutamine, 1 mM Na Pyruvate, 5 µg/ml Insulin, 5 µg/ml *N*-acetyl-l-cysteine, 1× Trace Elements B, 10 ng/ml d-Biotin, 100 µg/ml BSA, 100 µg/ml apo-Transferrin, 16 µg/ml Putrescine, 60 ng/ml Progesterone, 40 ng/ml Sodium Selenite 5 ng/ml Forskolin, 10 ng/ml CNTF, 1 ng/ml NT-3) supplemented with 10 ng/ml PDGF-AA. Proliferating OPCs were fed every 2–3 days and supplemented with 2–3× concentration of PDGF as needed to prevent OPC differentiation. After 6–7 days of proliferation, OPCs were harvested by trypsinization, plated into PDL-coated 96-well Falcon plates and centrifuged at 200×*g* to facilitate cell attachment, survival, and even distribution of cells.

### Oligodendrocyte protection assay

Expanded OPCs were plated at 10,000 cells/well in differentiation media supplemented with 40 ng/ml T3 and incubated for 24 h at 37 °C, 10 % CO_2_ prior to addition of test compounds. Adjusted concentrations of NCC library compounds were added to triplicate wells to account for toxicity. One hour after addition of test compounds, 10 Units/ml IFNγ + 1 ng/ml TNFα were added and cultures were incubated for 48 h. Controls were added in six replicate wells, negative control = 0.1 % DMSO; positive controls = no insults (no IFNγ and no TNFα) or 1.1 μM QTP. A 1:10 dilution of AB was added to each well, incubated 4 h at 37 °C, 10 % CO_2_ and fluorescence measured as above. In some experiments, following AB quantification, cells were fixed with 4 % paraformaldehyde and immunostained for MBP expression as described below.

### Immunofluorescence staining and imaging

Following compound treatment, media was removed leaving 50 μl/well using an ELx405 microplate washer (BioTek, Winooski, VT, USA), the automated plate washer was also used for all subsequent washing steps during the staining. Cells were fixed for 14 min with 4 % paraformaldehyde at room temperature (RT). Following fixation, plates were washed with 1 ml PBS leaving 50 μl/well behind. Cells were then incubated in blocking buffer (10 % normal goat serum (NGS) diluted in antibody buffer: 150 mM NaCl, 50 mM Tris Base, 1 % BSA, 100 mM l-lysine, 0.04 % sodium azide, pH 7.4), with 0.4 % Triton X-100 for 1 h at RT, then stained overnight at 4 °C with rat anti-MBP antibodies (Additional file 4: Table S3) diluted in blocking buffer with 0.08 % Triton X-100. The cells were washed and incubated with secondary antibodies and 0.3 μM DAPI for 1 h at RT. After a final wash, 100 μl of PBS was added to each well and plates imaged. Secondary antibody alone controls showed little to no background staining. Images were captured with a Nikon Eclipse TE-2000-U microscope, Zyla cMOS megapixel camera (ANDOR Technology, Belfast, UK), fitted with an automated stage controlled by NIS Elements AR software 4.0 (Melville, NY, USA). An air 10× lens was used to capture 2 images per well with 16 bit resolution, 2560 × 2160 pixels. Images for each assay run were captured using identical camera settings. Images were exported as TIFF files for analysis and quantification.

### Image quantification

TIFF files were analyzed using custom algorithms created with IN Cell Investigator Developer Toolbox (GE Health Sciences, Piscataway, NJ, USA). For cytokine protection assay, two images were analyzed per well and the data from triplicate wells was combined and averaged (total of six images per test condition). The overall amount of MBP expression/cell was quantified in each image by calculating the density × area of MBP staining, and normalizing to the total number of DAPI-stained nuclei in the same field. The calculated extent of MBP expression (OL differentiation) was then scaled to internal plate negative (0.1 % DMSO, set to 0) and positive controls (40 ng/ml T3 set to 100 %) to generate percent OL differentiation scores relative to the efficacy of positive control. For the cytokine protection assay, the positive controls were either no insult or 1.1 μM QTP. This analysis consistently generated quantitatively similar results when scaled to internal plate negative and positive control wells.

### Relative EC_50_ analysis

EC_50_ values were obtained by fitting the data to a sigmoidal dose–response curve-fitting function (Prism, GraphPad, San Diego, CA, USA). Serial dilutions of eight to ten different concentrations with three or four data points per concentration were used for curve fitting. Experiments were repeated at least two times.

### Statistical methods

For all experiments, assuming normal distribution, two-tailed t tests were used to evaluate comparisons between two groups and ANOVA was used when more than two groups were compared. For the quantitative analysis of in vitro differentiation, ANOVA with Bonferroni or Dunnett correction was used.

## Additional files

10.1186/s13104-016-2219-8 Acute OL differentiation hit compounds tested in the IFNγ protection assay.
10.1186/s13104-016-2219-8 Viability and OL differentiation EC_50_ curves for acute OL differentiation hits in cytokine protection assay.
10.1186/s13104-016-2219-8 NCC library cytokine protection screen data set.
10.1186/s13104-016-2219-8 Antibodies.

## Abbreviations

CNS: central nervous system
MS: multiple sclerosis
OL: oligodendrocytes
OPCs: oligodendrocyte precursor cells
DIV: days in vitro
MBP: myelin basic protein
DMSO: dimethylsulfoxide
HTS: high throughput screening
NCC: NIH clinical collection
