# Supplementary material for: Development of a high throughput drug screening assay to identify compounds that protect oligodendrocyte viability and differentiation under inflammatory conditions
Source: BMC Res Notes. 2016 Sep 5;9:444. doi: 10.1186/s13104-016-2219-8 (PMC5024459; doi:10.1186/s13104-016-2219-8)
Supplement: Supplementary file 1 — 10.1186/s13104-016-2219-8 Acute OL differentiation hit compounds tested in the IFNγ protection assay. [file 13104_2016_2219_MOESM1_ESM.pdf]

Table S1. Acute OL differentiation hit compounds tested in the IFNγ protection assay.

Compound	Drug Class	Viability (AB) EC_{50} [μM]	OL diff (MBP) EC_{50} [μM]
Raloxifene	Hormone	0.036	0.0034
Toremifene		0.049	0.014
Tamoxifen		0.039	0.017
Perphenazine	Tricyclic anti-depressants	0.057	0.035
Fluphenazine		0.041	0.012
Prochlorperazine		0.049	0.016
Trifluoperazine		0.054	0.015
Quetiapine		0.13	0.074
Perospirone	Non-Tricyclic anti-depressants	0.12	0.012
Nitalapram/Citalopram		0.52	-
Escitalopram		0.66	0.54
Metylperon		4.2	1.3
Bupropion		7.1	1.3
Clemastine fumarate	Muscarinic	0.053	0.44
Cogentin/Benztropine		0.014	0.04
Donepezil		0.53	0.3
Vesamicol		2.9	0.019
Oxybutynin		0.37	0.13
Ipratropium		0.23	0.028
Salmeterol	Adrenergic	0.28	0.075
Betaxolol		2.2	0.19
Esmolol		-	-
Ifenprodil	Ion Channel	0.4	0.24
Benproperine		0.52	0.072
Proxymetacaine		2	0.094
Dofetilide		-	-
DMPP**		160	0.89
Bifonazole	Anti-fungal	0.46	0.13
Clotrimazole		0.66	-
Ketoconazole		0.43	-

**Dimethylphenylpiperazinium
