# Supplementary material for: Development of a high throughput drug screening assay to identify compounds that protect oligodendrocyte viability and differentiation under inflammatory conditions
Source: BMC Res Notes. 2016 Sep 5;9:444. doi: 10.1186/s13104-016-2219-8 (PMC5024459; doi:10.1186/s13104-016-2219-8)
Supplement: Supplementary file 2 — 10.1186/s13104-016-2219-8 Viability and OL differentiation EC50 curves for acute OL differentiation hits in cytokine protection assay. [file 13104_2016_2219_MOESM2_ESM.pdf]

Fig. S1

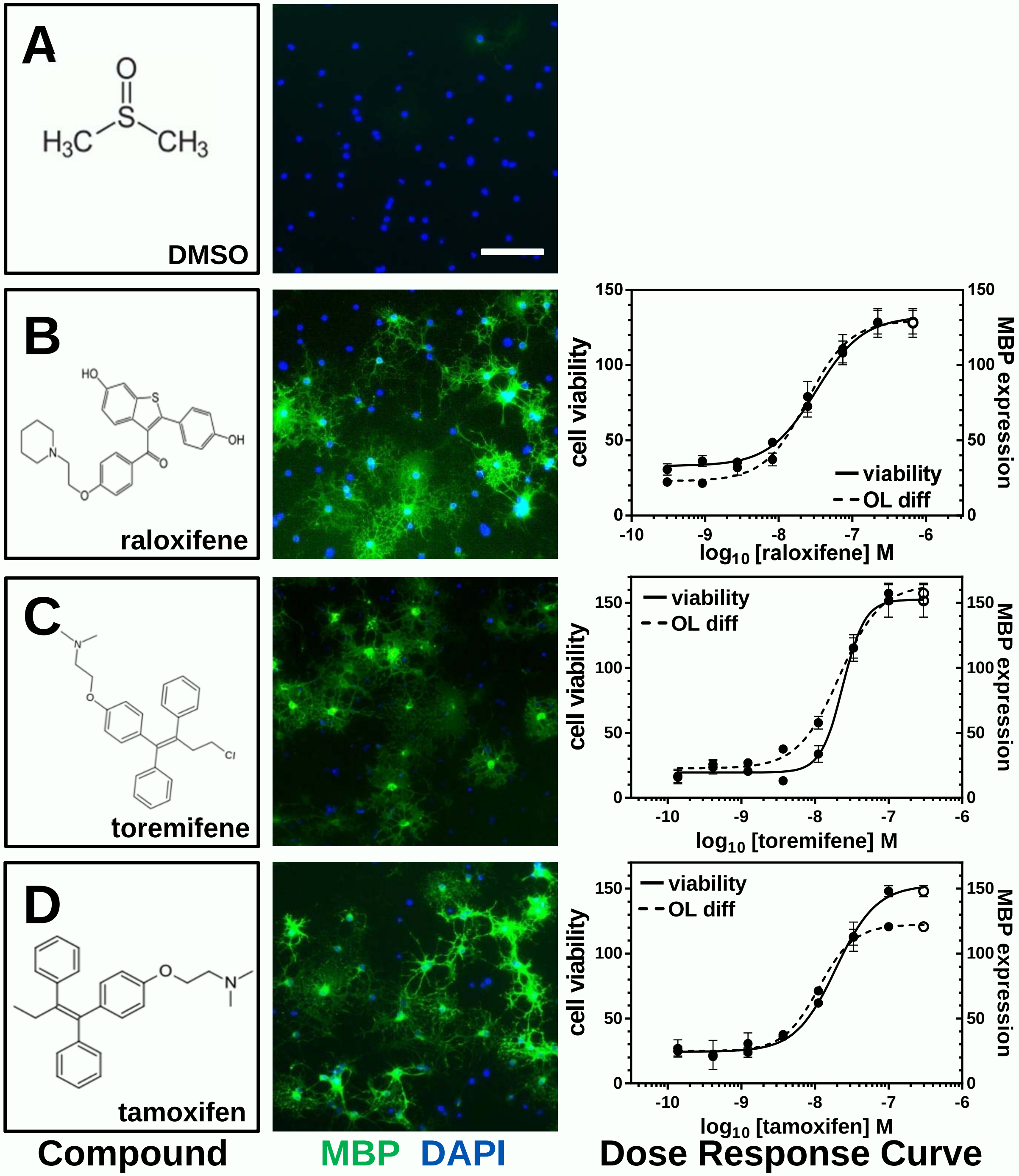

A
DMSO
B
raloxifene
C
toremifene
D
tamoxifen
cell viability
MBP expression
viability
OL diff
log10 [raloxifene] M
log10 [toremifene] M
log10 [tamoxifen] M
Compound
MBP DAPI
Dose Response Curve

Fig. S1

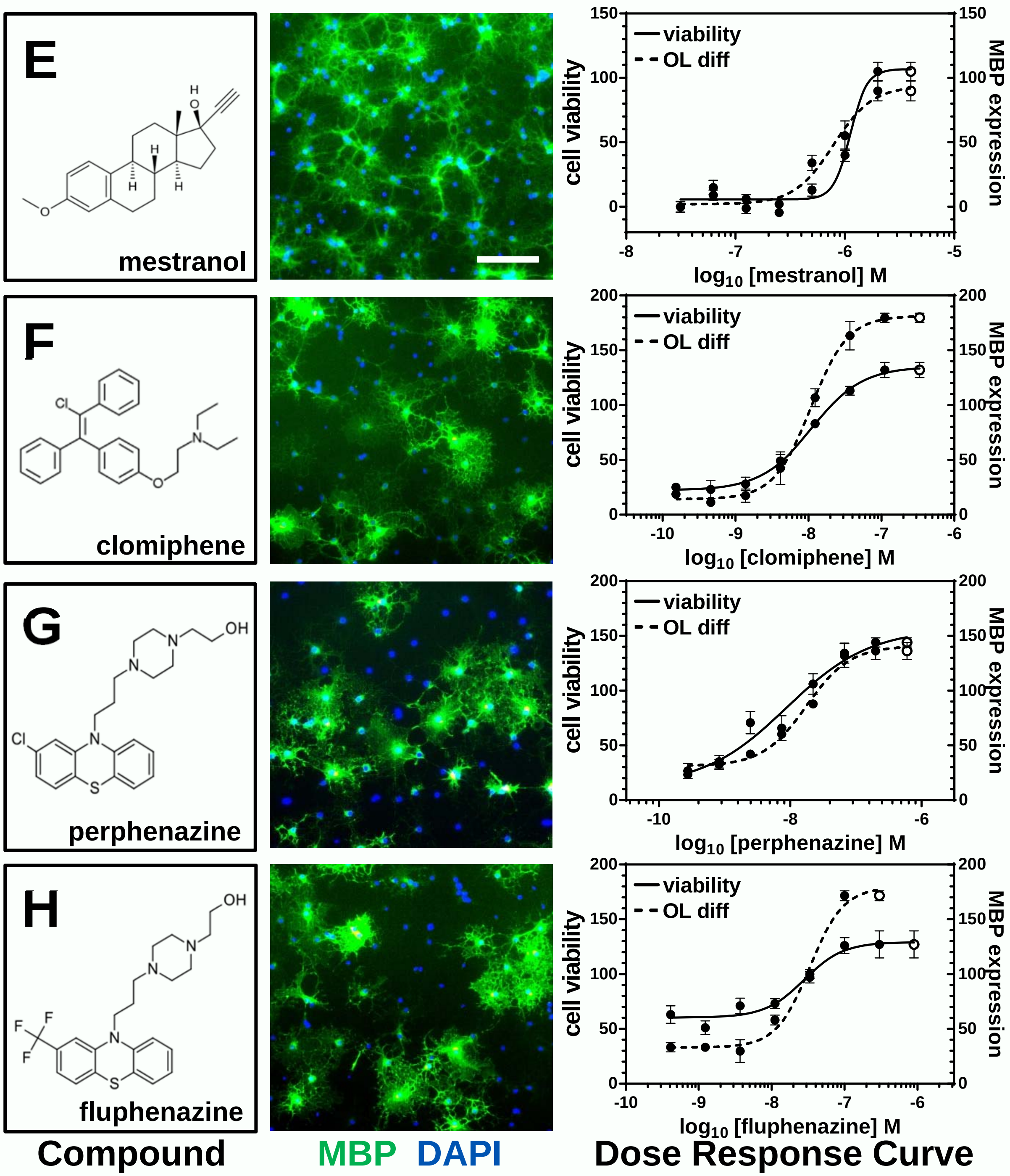
E
mestranol
F
clomiphene
G
perphenazine
H
fluphenazine
viability
OL diff
cell viability
MBP expression
log10 [mestranol] M
log10 [clomiphene] M
log10 [perphenazine] M
log10 [fluphenazine] M
Compound
MBP DAPI
Dose Response Curve

Fig. S1

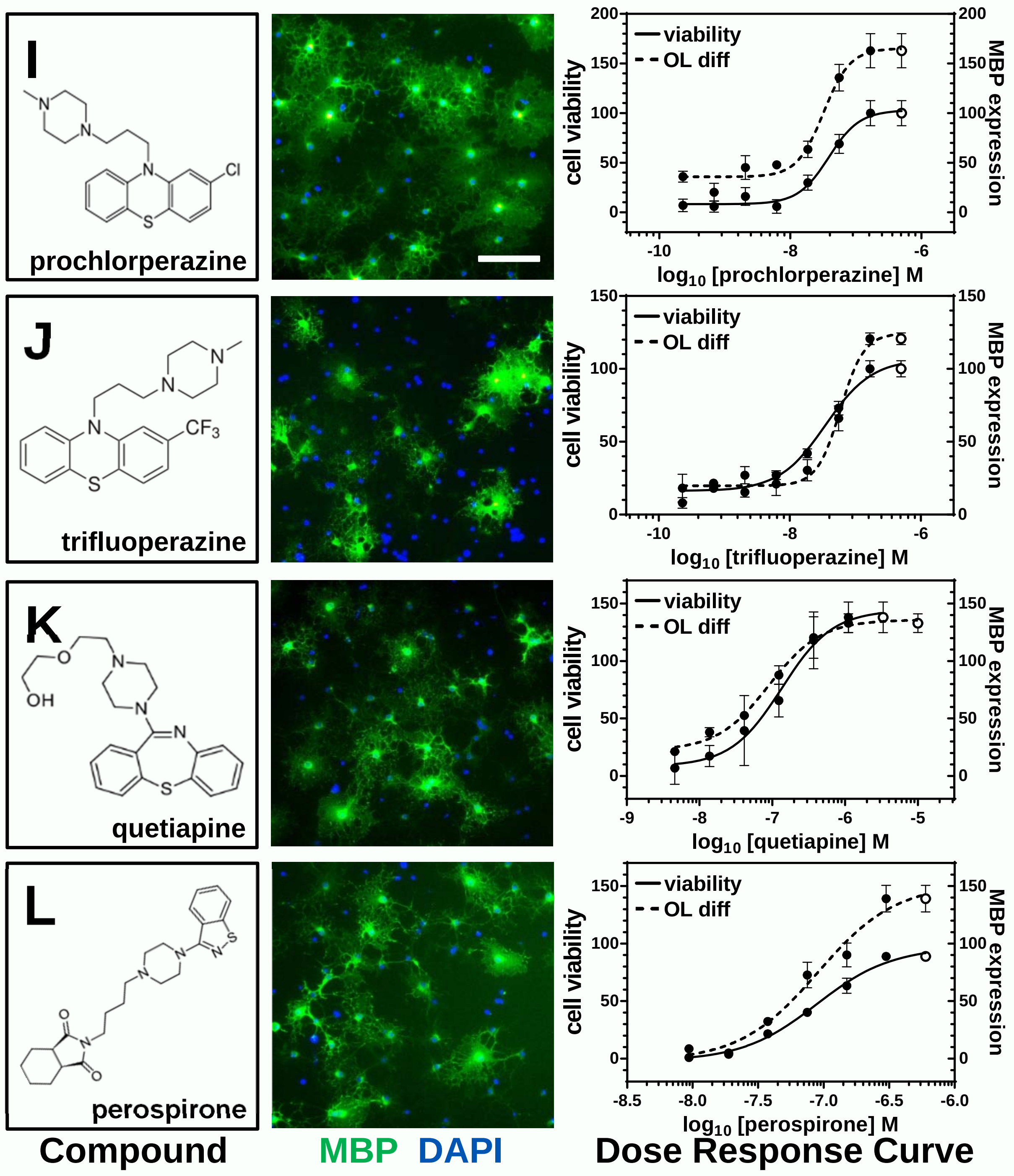

I
Cl
N
S
prochlorperazine
J
CF3
trifluoperazine
K
OH
quetiapine
L
perospirone
viability
OL diff
cell viability
MBP expression
log10 [prochlorperazine] M
log10 [trifluoperazine] M
log10 [quetiapine] M
log10 [perospirone] M
Compound
MBP DAPI
Dose Response Curve

Fig. S1

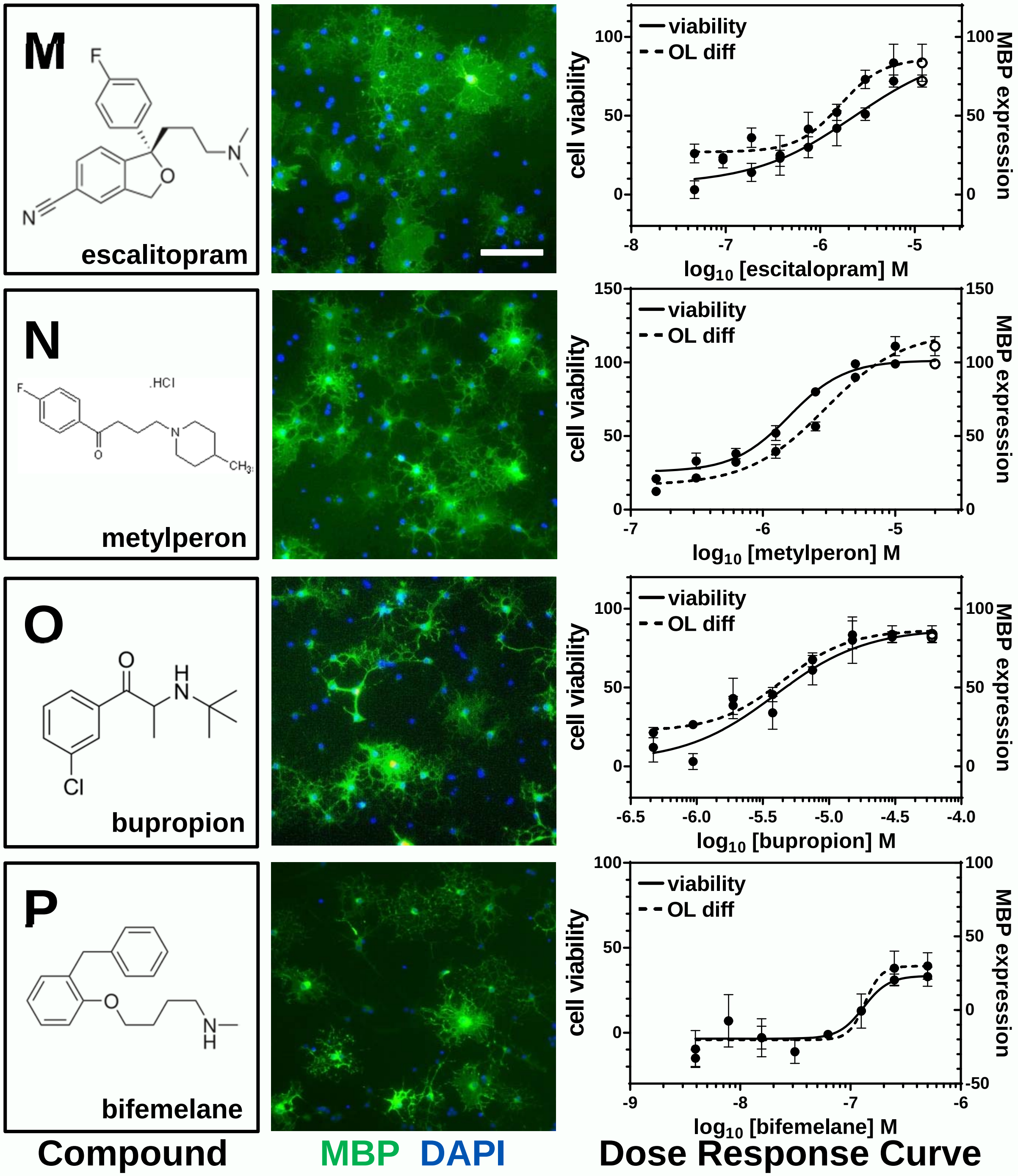

M
F
N
O
escalitopram
viability
OL diff
cell viability
MBP expression
log10 [escitalopram] M
N
.HCl
F
O
N
CH3
metylperon
log10 [metylperon] M
O
O
H
N
Cl
bupropion
log10 [bupropion] M
P
O
N
H
bifemelane
log10 [bifemelane] M
Compound
MBP DAPI
Dose Response Curve

Fig. S1

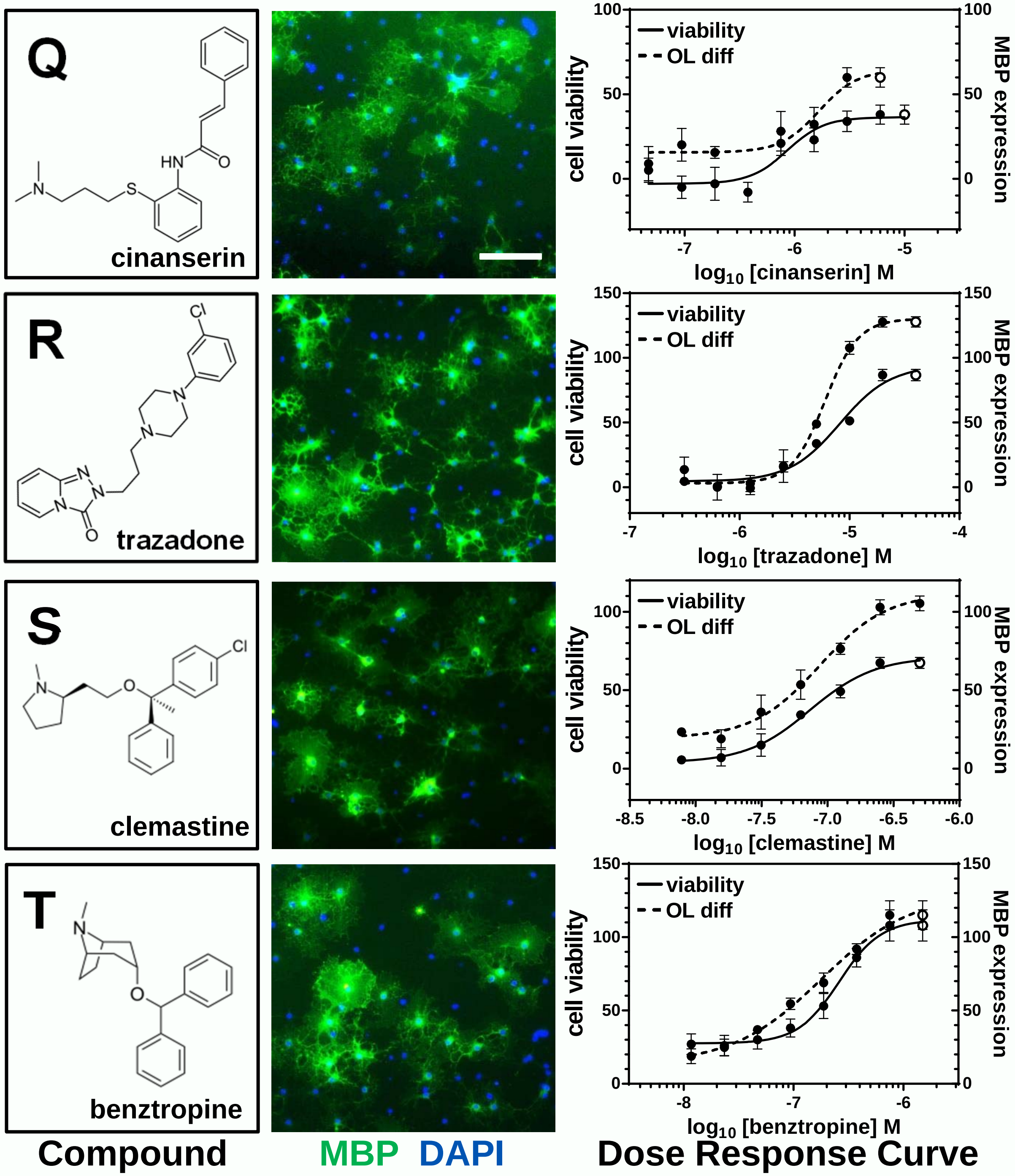

Q
cinanserin
R
trazadone
S
clemastine
T
benztropine
viability
OL diff
cell viability
MBP expression
log10 [cinanserin] M
log10 [trazadone] M
log10 [clemastine] M
log10 [benztropine] M
Compound
MBP DAPI
Dose Response Curve

Fig. S1

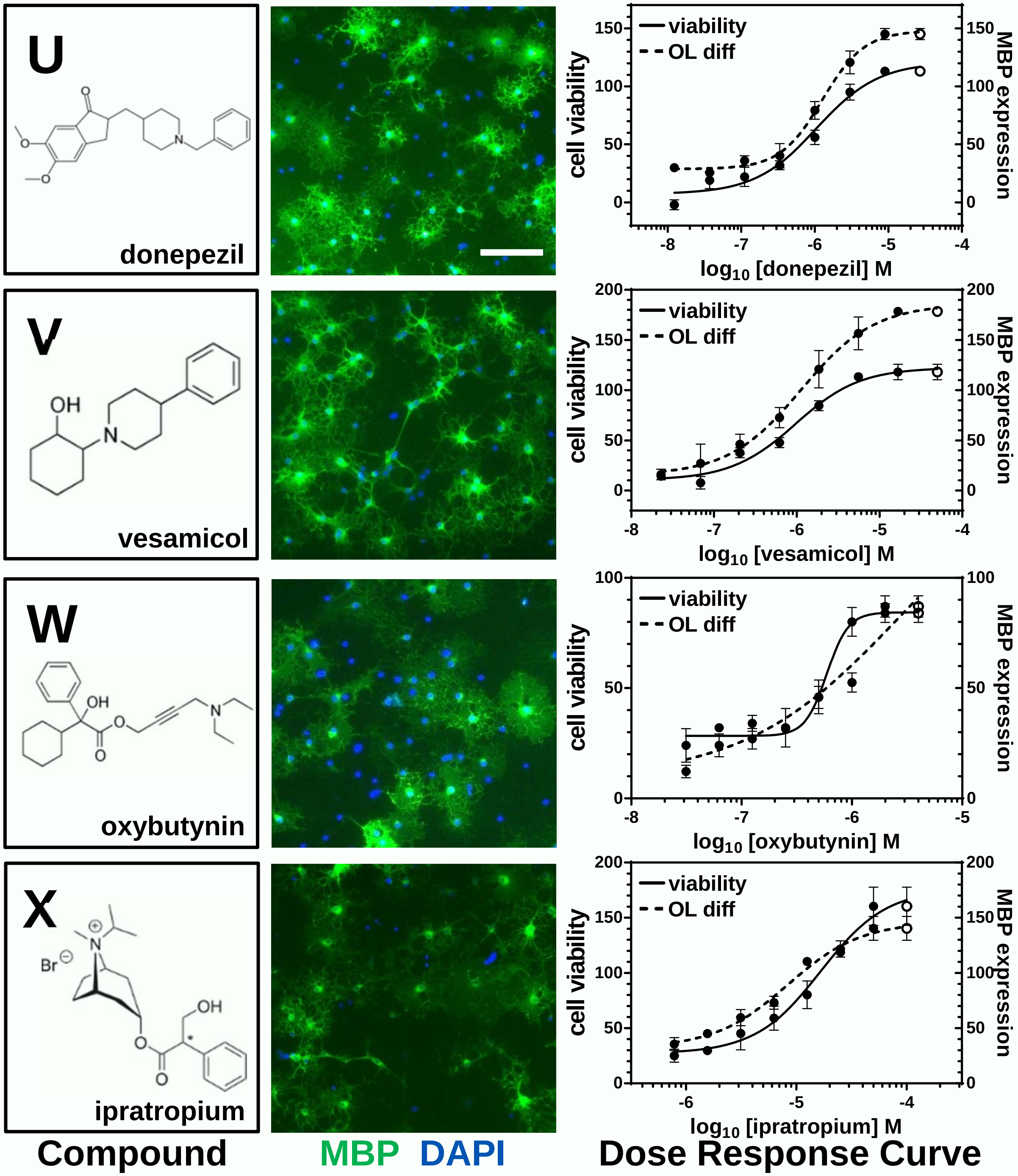

U
donepezil
V
OH
N
vesamicol
W
OH
N
O
oxybutynin
X
Br
N
OH
O
ipratropium
viability
OL diff
cell viability
MBP expression
log10 [donepezil] M
log10 [vesamicol] M
log10 [oxybutynin] M
log10 [ipratropium] M
Compound
MBP DAPI
Dose Response Curve

Fig. S1

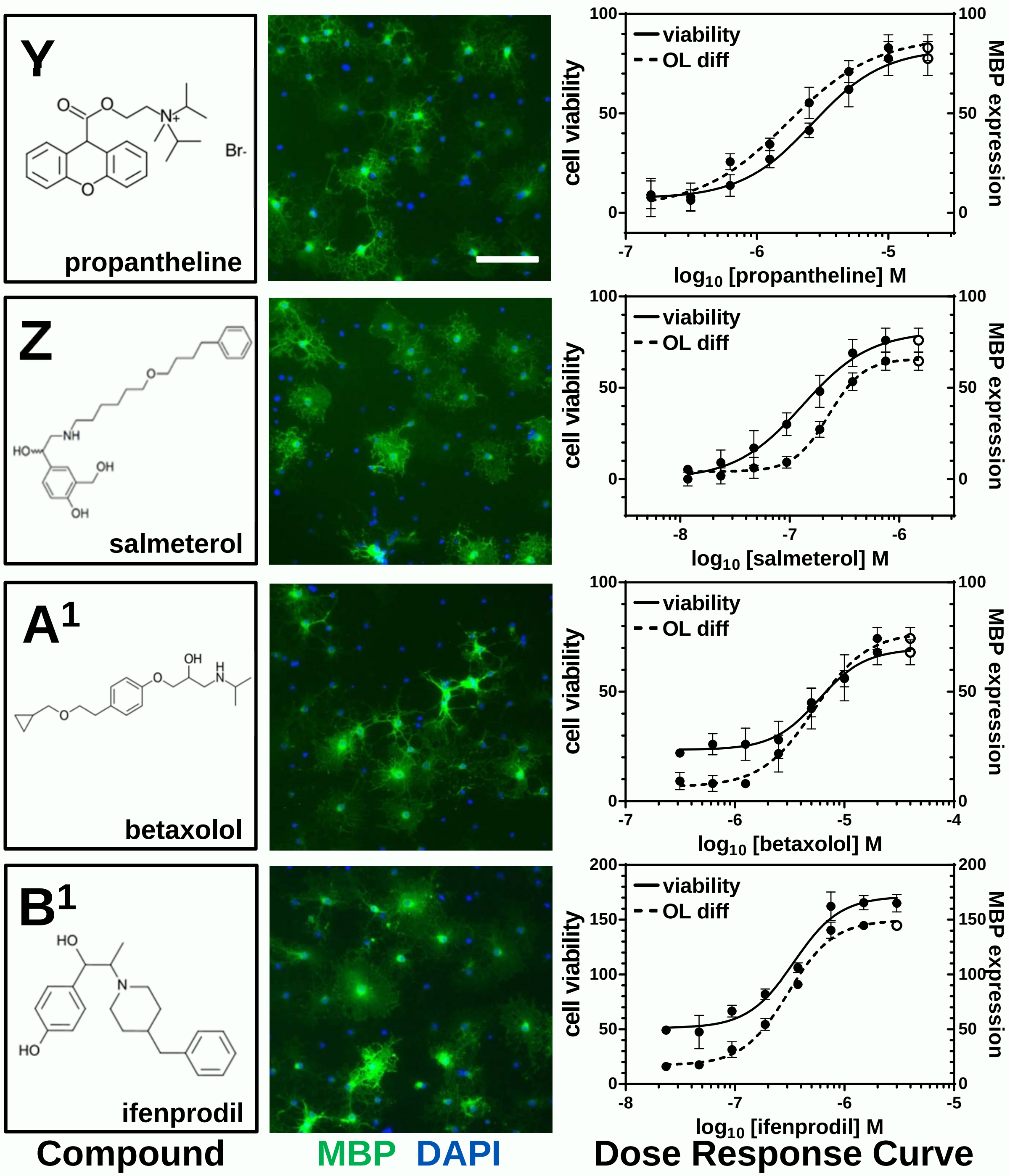

Y
propantheline
Z
salmeterol
A1
betaxolol
B1
ifenprodil
Compound
MBP DAPI
Dose Response Curve
viability
OL diff
cell viability
MBP expression
log10 [propantheline] M
log10 [salmeterol] M
log10 [betaxolol] M
log10 [ifenprodil] M

Fig. S1

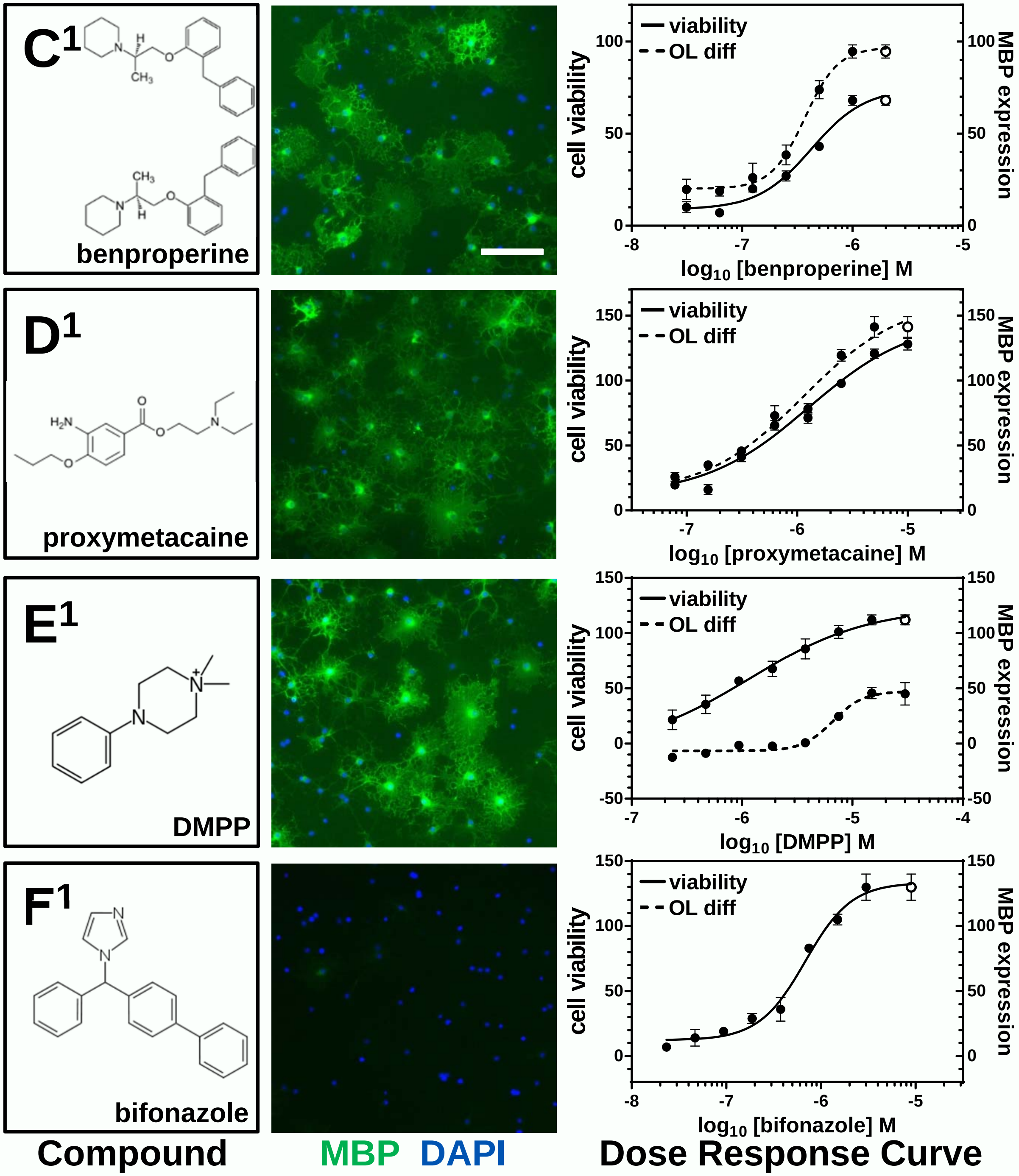
C1
benproperine
cell viability
MBP expression
viability
OL diff
log10 [benproperine] M
D1
proxymetacaine
log10 [proxymetacaine] M
E1
DMPP
log10 [DMPP] M
F1
bifonazole
log10 [bifonazole] M
Compound
MBP DAPI
Dose Response Curve

Fig. S1

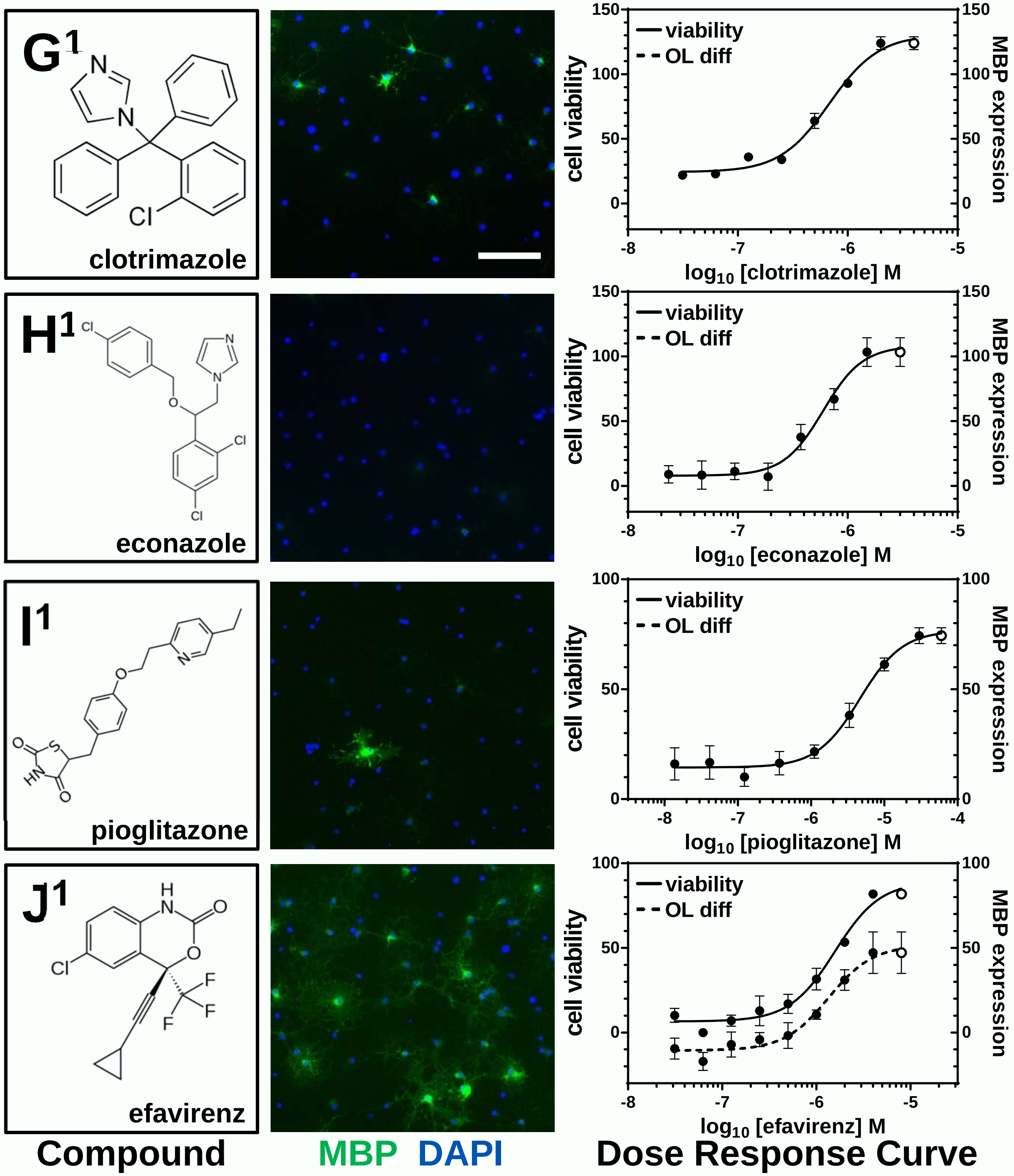
G1
clotrimazole
H1
econazole
I1
pioglitazone
J1
efavirenz
Compound
MBP DAPI
Dose Response Curve
cell viability
MBP expression
viability
OL diff
log10 [clotrimazole] M
log10 [econazole] M
log10 [pioglitazone] M
log10 [efavirenz] M

Fig. S1

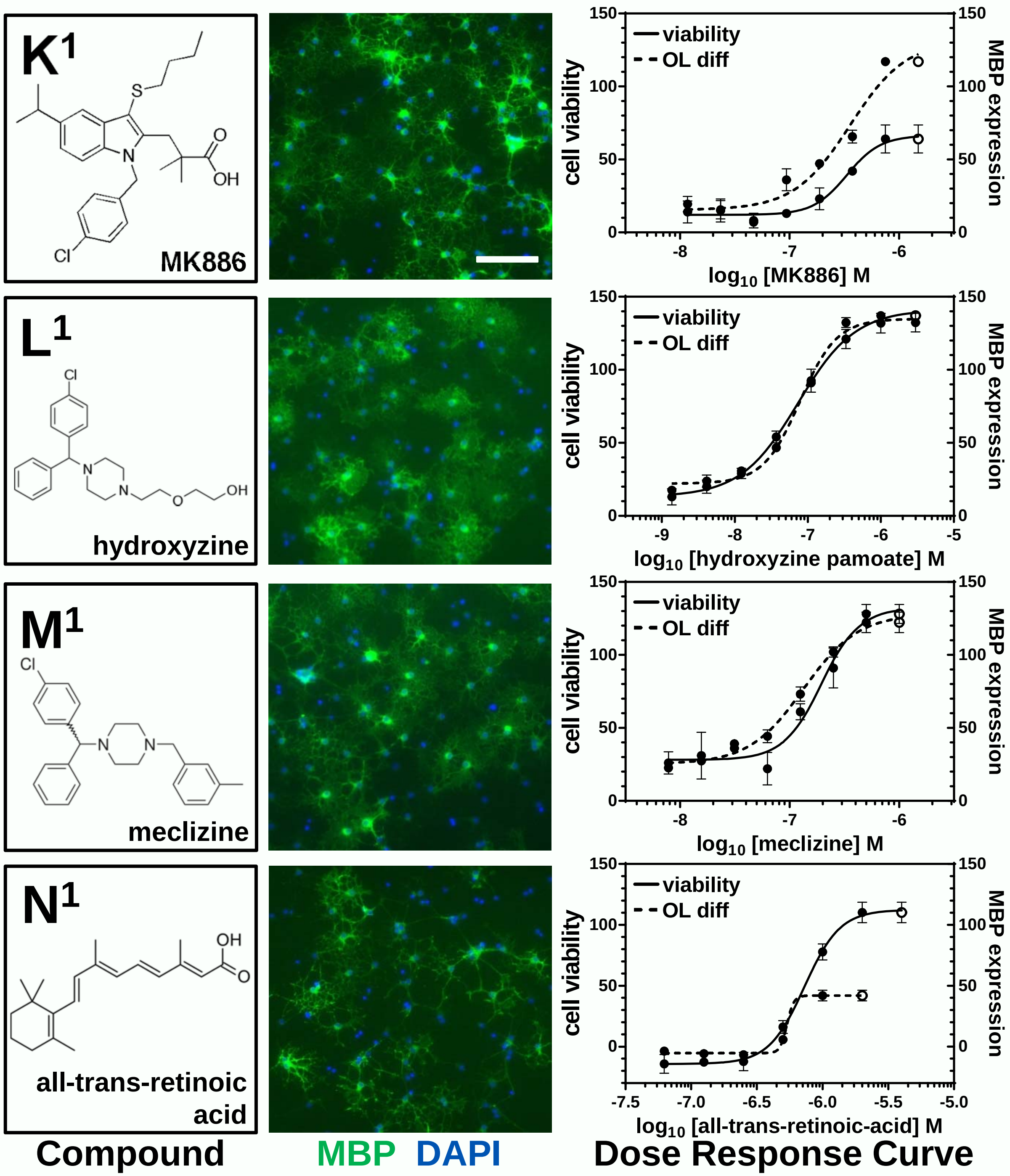
K1
MK886
L1
hydroxyzine
M1
meclizine
N1
all-trans-retinoic acid
viability
OL diff
cell viability
MBP expression
log10 [MK886] M
log10 [hydroxyzine pamoate] M
log10 [meclizine] M
log10 [all-trans-retinoic-acid] M
Compound
MBP DAPI
Dose Response Curve

Fig. S1

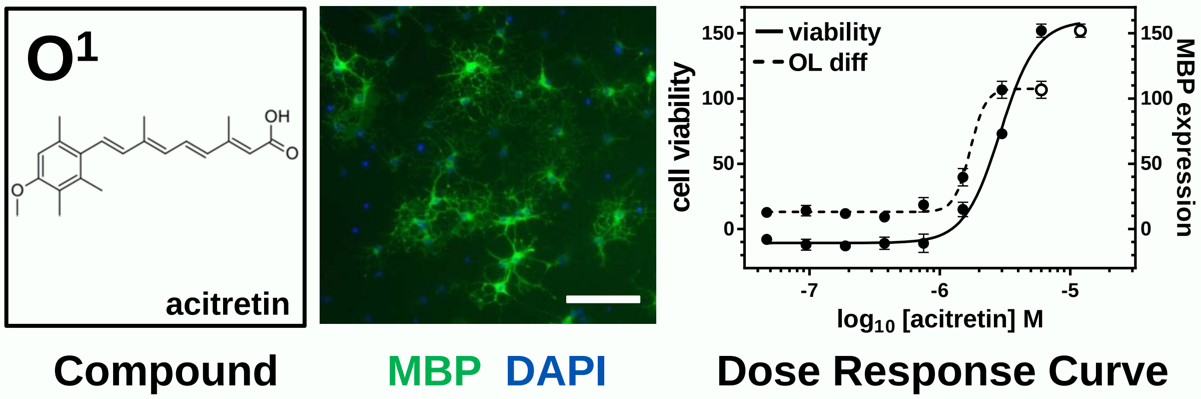

Figure S1. Structures, images, and EC_{50}s of cytokine protection hits. A-O[1] first column, Chemical structure and name of each hit compound and 0.1% DMSO. A-O[1] second column, Example image of each hit compound at the most efficacious concentration showing MBP (green) and DAPI (blue) staining. Bar = 100 µM. A-O[1] third column, Representative dose-response curves of viability (solid lines) and OL differentiation (dashed lines) in cytokine protection assay for each hit compound. Open circles indicate points added to round off the curve. Note that no OL differentiation dose response curves are shown for the bifonazole, clotrimazole, econazole, and piaglitazone because they rescued survival but not MBP expression.
